# Applications of Artificial Intelligence in Medical Education: A Systematic Review

**DOI:** 10.7759/cureus.79878

**Published:** 2025-03-01

**Authors:** Eric Hallquist, Ishank Gupta, Michael Montalbano, Marios Loukas

**Affiliations:** 1 Department of Family Medicine, Prevea Shawano Avenue Health Center, Green Bay, USA; 2 Department of Anatomical Sciences, St. George’s University School of Medicine, St. George, GRD; 3 Department of Clinical Anatomy, Mayo Clinic, Rochester, USA

**Keywords:** machine learning algorithms, medical education assessment, medical examination, natural language processing models, teaching undergraduate and postgraduate

## Abstract

Artificial intelligence (AI) models, like Chat Generative Pre-Trained Transformer (OpenAI, San Francisco, CA), have recently gained significant popularity due to their ability to make autonomous decisions and engage in complex interactions. To fully harness the potential of these learning machines, users must understand their strengths and limitations. As AI tools become increasingly prevalent in our daily lives, it is essential to explore how this technology has been used so far in healthcare and medical education, as well as the areas of medicine where it can be applied. This paper systematically reviews the published literature on the PubMed database from its inception up to June 6, 2024, focusing on studies that used AI at some level in medical education, following the Preferred Reporting Items for Systematic Reviews and Meta-Analyses guidelines. Several papers identified where AI was used to generate medical exam questions, produce clinical scripts for diseases, improve the diagnostic and clinical skills of students and clinicians, serve as a learning aid, and automate analysis tasks such as screening residency applications. AI shows promise at various levels and in different areas of medical education, and our paper highlights some of these areas. This review also emphasizes the importance of educators and students understanding AI's principles, capabilities, and limitations before integration. In conclusion, AI has potential in medical education, but more research needs to be done to fully explore additional areas of applications, address the current gaps in knowledge, and its future potential in training healthcare professionals.

## Introduction and background

Artificial intelligence (AI) refers to technologies designed to mimic human intelligence, and its applications have evolved significantly over the years. Initially, AI was used in narrow fields like digital searching and facial recognition and for tasks such as analyzing cardiac rhythms [1,2]. However, recent advancements in natural language processing (NLP) have greatly expanded AI's capabilities, enabling systems to engage in more complex tasks, such as having conversations in human language [3,4]. When combined with machine learning (ML) algorithms, which allow AI to process large amounts of data and make decisions based on patterns, these systems have the potential to perform tasks autonomously with minimal human input [5]. In medical education, these developments have opened up new opportunities for AI to support learning in various ways. Generative AI models, like Chat Generative Pre-Trained Transformer (ChatGPT; OpenAI, San Francisco, CA), are increasingly being used to answer student queries, create personalized lessons, provide tailored feedback, and even facilitate simulations of virtual patients based on aggregated data [6,7]. In clinical practice, AI is already making an impact, such as using automated systems that analyze heart rhythms with defibrillators [8]. As AI tools gain popularity and adoption in healthcare and education, it becomes crucial for medical educators to comprehend how these tools integrate into their curricula [9-11]. However, to fully capitalize on these technologies, it is essential that medical educators and students understand the strengths and limitations of AI tools. This paper aims to provide a systematic review of how AI is currently being applied in medical education. It focuses on studies that assess AI's role across different educational stages, from medical school to postgraduate training and continuing medical education (CME). The goal of this study is not only to highlight the positive contributions of AI in medical education so far but also to identify gaps in knowledge and encourage further research to explore the full potential of AI in training healthcare professionals.

Materials and methods

Search Strategy

In this systematic review, the authors adhered to the Preferred Reporting Items for Systematic Reviews and Meta-Analyses guidelines. Two independent researchers (E.H. and I.G.) conducted a literature search on the PubMed database using the keywords "artificial intelligence" AND "medical education" from inception up to June 6, 2024, with a third researcher (M.M.) involved to reach a consensus when needed. The practice of backward citation was also used to locate additional articles. This was done by reviewing the references or citations within an article from the search synthesis to find studies relevant to our research topic. Only PubMed was used because it is a repository for clinical medicine and medical education; other databases or search tools, such as Scopus, Web of Science, or Google Scholar, were not used, as their literature is not limited to biomedical, clinical, or medical sciences, which was our main focus. The search strategy registration was not required by our institution, as our study did not involve human subjects.

Selection Criteria

Articles were selected based on the following inclusion criteria: 1) article discussion of medical education at the level of medical school, postgraduate training, or CME; 2) discussion of AI, including any subset of AI such as ML models, generative AI large language models (LLMs), or NLP models; 3) reporting of numerical data relevant to AI in medical education; and 4) text available in the English language. The exclusion criteria for articles were as follows: 1) discussion of medical education without mentioning AI; 2) discussion of AI without mentioning medical education; 3) discussion of neither AI nor medical education; 4) letters, editorials, expert opinions, and other descriptive reports; and 4) unpublished studies or preprints.

## Review

Results

**Figure 1:**
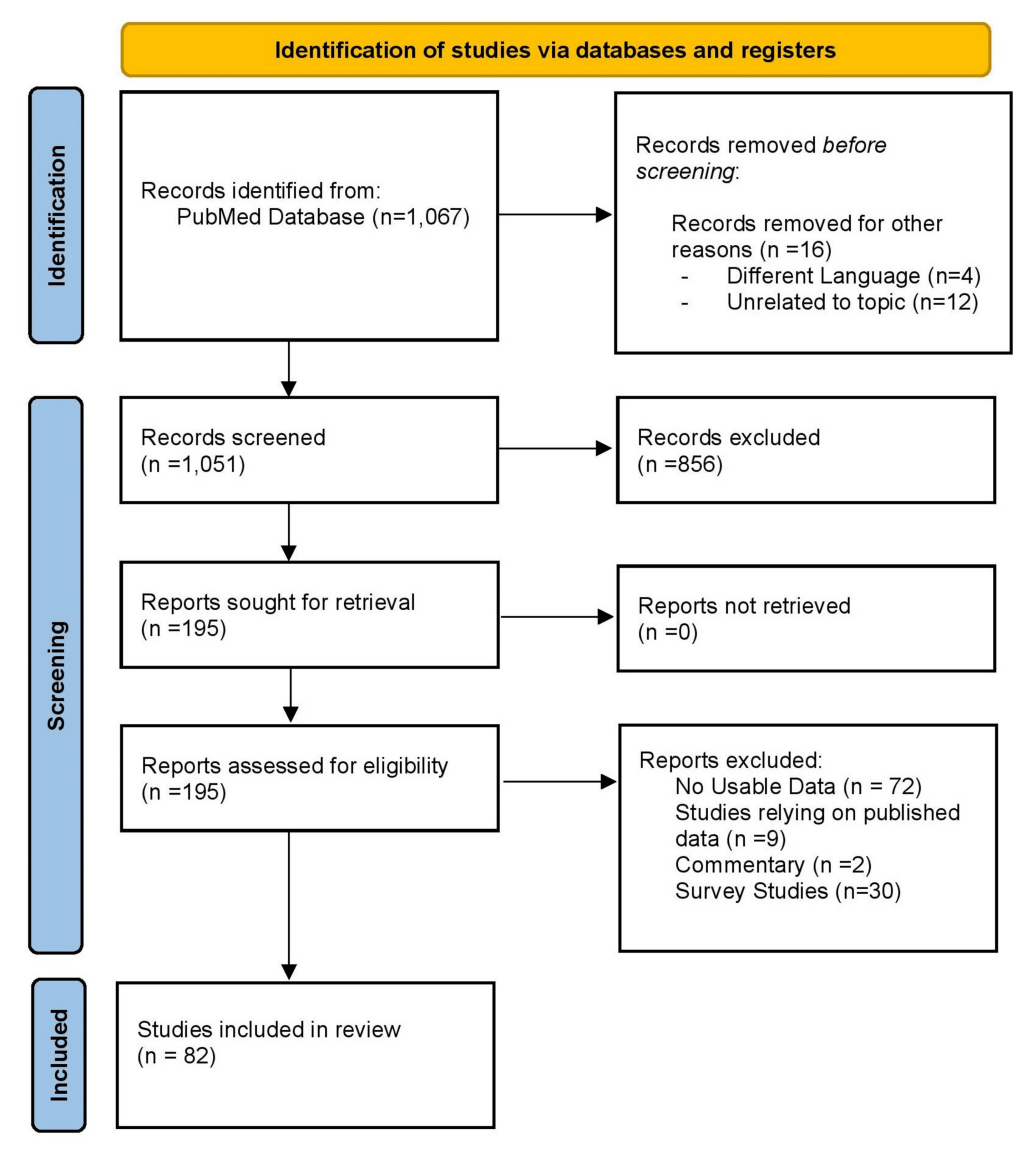
The PRISMA flowchart PRISMA: Preferred Reporting Items for Systematic Reviews and Meta-Analyses Image credit: This is an original image created by the author Ishank Gupta

The initial search of the PubMed database yielded 781 articles, with another 286 identified through backward citation searching. After applying inclusion and exclusion criteria to the 1,067 articles, 195 articles were sought for retrieval, and all 195 articles were retrieved. After retrieval, 113 reports were further excluded after a review of the entire text, thereby leaving 82 articles for final inclusion in this review (Figure 1).

Assessing AI’s Clinical Knowledge and Decision-Making Through Standardized Assessments

Fourteen studies employed the LLM ChatGPT as their AI tool of choice [12-25]. Among these, six studies directly compared the performance of GPT-3.5 and GPT-4.0 [12,18,20,22-24], with GPT-4 consistently demonstrating superior performance over GPT-3.5. The model was evaluated across various disciplines of medicine: surgery [12], parasitology [13], microbiology [14], neurology [18], and bioethics [21]. Three studies compared ChatGPT's performance with human test-takers [13,16,24]. Eight studies assessed ChatGPT's performance on national medical board exams [15,17,19,20,22-25], including the German medical licensing examination [24], the European Exam in Core Cardiology [15], the Family Medicine Board Exam in Taiwan [17], and the United States Medical Licensing Examination (USMLE) Step 3 study question bank [23]. Some studies did not specify the ChatGPT version used [17], and in four studies, questions in languages other than English were utilized [17,20,24,25]. A common limitation across these studies was ChatGPT's inability to interpret questions involving tables, graphs, or images, restricting evaluations to text-based questions only.

Using AI Tools to Craft Medical Illness Scripts and Exam Questions

Several studies utilized ChatGPT to generate medical examination questions and illness scripts. Yanagita et al. assessed whether ChatGPT-4 could create illness scripts for diseases according to Japanese medical education standards [26]. Another study aimed to compare script concordance tests (SCTs) generated by ChatGPT with those created by clinical experts [27]. Hudon et al. used a mixed-method approach, evaluating three SCTs generated by ChatGPT alongside three SCTs crafted by clinical experts and gathering feedback through a web-based survey from clinician-educators and resident doctors specializing in psychiatry in Quebec, Canada [27]. Klang et al. generated 210 multiple-choice questions (MCQs) using ChatGPT-4, and Cheung et al. used ChatGPT to generate 50 MCQs for medical examinations [28,29]. These studies are listed in Table 1.

**Table 1:** Overview and description of studies where AI tools were used to generate multiple choice medical examination questions and illness scripts AI: artificial intelligence; GPT: Generative Pre-Trained Transformer; SCTs: script concordance tests; MCQs: multiple-choice questions

Study	Study purpose	AI tool used	Number of questions/illness scripts	Study features	Results	Areas of errors in AI-generated content	Conclusions
Yanagita et al. [26]	Aims to investigate whether AI can generate illness scripts	GPT-4	184 disease illness scripts	Three physicians assessed each illness script using a three-tier grading scale	The illness scripts received “A,” “B,” and “C” ratings of 56.0% (103/184), 28.3% (52/184), and 15.8% (29/184), respectively	Cardiovascular and psychiatric systems had the highest number of “C” ratings	GPT-4 generates illness scripts rapidly and with high quality
Hudon et al. [27]	Comparing SCTs generated by ChatGPT with those produced by clinical experts	ChatGPT	Three ChatGPT-generated SCTs vs. three expert-created SCTs	102 educators evaluated the six SCTs based on three preset criteria	No significant distinctions between the two types of SCT scenario (p = 0.84)	-	ChatGPT is useful for developing educational materials
Klang et al. [28]	Writing multiple choice examination questions for medical students	GPT-4	210 MCQs generated by AI	AI-generated questions reviewed by specialist physicians. Physicians were blinded to the source of the questions	One question (0.5%) was defined as false; 15% of questions required revisions	Inaccurate medical terminology and inaccuracies in age-sensitivity, gender-sensitivity, and geographic sensitivity	GPT-4 can assist in creating multiple-choice medical exam questions
Cheung et al. [29]	Comparison of graduate medical exam MCQs: University Professors vs. ChatGPT-generated	ChatGPT	50 MCQs generated by ChatGPT and 50 MCQs drafted by two university professors	Evaluated by five independent assessors. Scoring is based on a standardized system	No significant difference in question quality among the groups	In the relevance domain, the AI was inferior to humans	ChatGPT can generate MCQs for medical graduate exams

AI as a Learning Aid in Medical School, Residency Training, and CME

Thirty publications examined the role of AI in enhancing medical education, encompassing a range of applications. Fifteen studies focused on using AI as a teaching tool for medical students [30-44]. Nine explored AI's applications in residency training [35,36,45-51], and six investigated AI's potential role in CME [31,35,36,52-54]. Price et al. proposed an ML model to enhance outcomes in medical specialty board recertification among physicians [54]. Six studies suggested that AI can improve training in diagnosis, communication, and radiograph interpretation for medical professionals [38,43-45,48,52]. These studies are listed in Table 2.

**Table 2:** Studies showing AI as a learning aid CME: continuing medical education; AI: artificial intelligence; OSCE: objective structured clinical examination; VOA: virtual operative assistant; SVMs: support vector machines; COMET: collaborative intelligent tutoring system; NLP: natural language processing; VICEE: Virtual Clinical Encounter Examination; CBCF: content-boosted collaborative filtering

Study	Study group	Control group present	Study summary
Monlezun et al. [30]	Medical students	No	Machine learning-enhanced causal inference analysis of a multisite cohort to boost medical trainees' skills in counseling patients about nutrition and also improve their own dietary habits
Ruberto et al. [31]	Medical students and postresidency or CME	No	A simulation platform that adjusts to a participant's cognitive load in real-time, offering potential for advancing expertise in resuscitation medicine
Yang and Shulruf [32]	Medical students	Yes	The addition of expert-led + AI-assisted tutoring to the surgical curriculum showed potential benefits, with the expert-led + AI group outperforming and improving more in the end-of-surgical block OSCE compared to the expert-led group
Nakawala et al. [33]	Medical students	Yes	A context-aware software framework for thoracocentesis training in surgical workflows yielded results comparable to traditional mentor-based training
Fazlollahi et al. [34]	Medical students	No	A randomized clinical trial showed that VOA feedback led to better performance and skill transfer than remote expert instruction in surgical training
Allen et al. [35]	Medical students, residents, and postresidency or CME	No	SVMs offer more accurate predictions of competency in laparoscopic training tasks than conventional methods of evaluation
Mirchi et al. [36]	Medical students, residents, and postresidency or CME	No	The virtual operative assistant, an AI tool, accurately classified skilled and novice participants, highlighting the potential of integrating AI and virtual reality simulation into surgical education
Del Blanco et al. [37]	Medical students	Yes	A game-like simulation to improve novices' perceptions and performance during their first operating theater experience. The simulation was effective in reducing fears and errors, and enhancing perceived knowledge and collaboration among the students
Cheng et al. [38]	Medical students	Yes	An AI-based medical image learning system significantly enhanced medical students' ability to identify hip fractures on pelvic X-rays
Wang et al. [39]	Medical students	No	AIteach (AITEACH Limited, Cambridge, UK), a virtual case system using NLP and hospital records, generated clinical cases for medical students, improving their clinical thinking skills
Denny et al. [40]	Medical students	No	A novel electronic advisor system using NLP to identify geriatric medicine competencies by analyzing medical students' clinical notes. Such models can be used to assess specific competencies among trainees
Maicher et al. [41]	Medical students	No	A virtual standardized patient system that assesses students' history-taking skills by understanding, responding, and providing immediate feedback on their performance
Hamdy et al. [42]	Medical students	No	The online VICEE effectively assessed medical students' non-psychomotor clinical competencies
Suebnukarn and Haddawy [43]	Medical students	No	COMET, an intelligent tutoring system, assessed clinical reasoning in problem-based learning among students. Such clinical reasoning models can be combined with traditional tutoring strategies to effectively emulate human tutor hints
Woo et al. [44]	Medical students	No	An intelligent tutoring system capable of engaging in a natural language dialogue with a student
Merritt et al. [45]	Residents	No	An AI-driven simulation platform to evaluate and provide real-time feedback to residents on a standardized, simulated conversation
Bissonnette et al. [46]	Residents	No	Machine learning algorithms were used to assess surgical performance among trainees executing a virtual reality hemilaminectomy
Lin et al. [47]	Residents	No	A novel hybrid prediction algorithm, CBCF, predicts the difficulty level of each case to create personalized training programs for radiology trainees
Hershberger et al. [48]	Residents	No	The NLP model, ReadMI, was used to enhance motivational interviewing skills among residents to encourage patients to change high-risk lifestyle behaviors

NLP, Motion Analysis, Clinical Skills, and Building Competence in Stakeholders

Thirty-four studies examined ML's capabilities in automating analysis tasks rather than direct applications to medical education [55-88].

Studies using NLP: Two studies evaluated the clinical competency of residents using NLP in clinical assessments [57,58]. Seven investigated the use of AI to assess medical school or residency applications [63-69]. One paper explored the potential of AI in assisting with writing letters of recommendation [76]. One study used AI in a virtual Q&A session for fellowship applicants, finding that many applicants considered the Chatbot helpful [56]. One used NLP to evaluate medical students’ clinical notes to assess their exposure to the American Association of Medical College geriatric competencies [72]. Two reported on AI's role in analyzing feedback from clinical supervisors and its correlation with clinical entrustment decisions [74,75].

AI in motion analysis and clinical skills: Four studies used AI models to assess and evaluate clinical skill expertise via operational exposure and surgical hand movements [59-62]. Another focus is predicting correct answers based on students' eye movements while viewing whole slide images [71]. One study used ChatGPT-assisted training to enhance clinical skills among pediatric trainees [73], and three focused on AI in medical imaging [85-87]. Additionally, one study examined AI's ability to detect racial stereotypes in medical scenarios [70].

Building competency in AI among stakeholders: Twelve papers addressed building competence in stakeholders regarding AI. Of these, eight focused on teaching AI fundamentals at the medical school level and postgraduate training [77-84] and one on the current state of AI education in German universities [88]. Abid et al. proposed a one-month elective on AI and ML for fourth-year medical students [81]. One study used surveys to measure the use of ChatGPT among faculty members [55].

Discussion

Advancements of AI in Medical Education and Assessments

GPT-3.5 was launched in November of 2022, and GPT-4 was launched in March of 2023. GPT-4 has demonstrated significant advancements in accuracy and clinical reasoning compared to its predecessor, GPT-3.5, in medical assessments. Notably, while ChatGPT-3.5 failed the UK Radiology Fellowship Exam, ChatGPT-4 passed with a score of 75.5% [22]. Meyer et al. observed that while GPT-4 successfully passed all three German medical licensing examinations tested, GPT-3.5 only passed one out of three [24]. These improvements position GPT-4 as a more reliable tool for delivering precise responses to complex medical queries [12,18,24]. In other studies, ChatGPT achieved an accuracy rate of 76.4% in surgical subspecialties [12], 60.8% in parasitology [13], 80% in microbiology [14], 64% in neurology [18], and an accuracy rate of 59.6% in bioethics [21]. Chen et al. found that ChatGPT excelled in bioethical questions related to death and physician-patient relationships but faced challenges with topics like abuse and informed consent [21]. Therefore, current AI capabilities may not be consistent enough to handle complex ethical decisions in medicine autonomously. This necessitates and emphasizes the need for human oversight, especially in critical scenarios [21].

In several studies, AI tackled difficult medical licensing or subspecialty board exams, which consist of complex questions requiring years of preparation, deductive reasoning, and extensive knowledge. In one study by Friederichs et al., ChatGPT answered 65.5% of progress test questions correctly, surpassing German medical students in their first three years of study [16]. ChatGPT also achieved a passing score on the European Exam in Core Cardiology with an accuracy rate of approximately 60% [15] but failed the 2022 Family Medicine Board Exam in Taiwan with only 41.6% accuracy [17]. Additionally, ChatGPT performed well on the USMLE Step 3 study question bank, achieving an overall accuracy of 84.7% [23]. In two studies, ChatGPT outperformed human test-takers [16,24], whereas in one study, it performed less effectively [13]. This shows the evolving capacity of AI to comprehend and process intricate medical information in a very short period. However, there are varying performance outcomes across studies when handling questions in languages other than English, where the model's linguistic database is less robust [17,24,25]. Despite the challenges, ChatGPT, even in its beta version, achieved satisfactory or high percentile scores, highlighting its growing capabilities in medical knowledge [15,19,20,22-25]. However, there is a need for dedicated research to better understand the factors influencing AI's test performance, including language disparities, and to determine whether the assessed knowledge is universally applicable or specific to a particular country [89]. Close collaboration between AI developers and medical professionals will be essential to ensure the effectiveness of these tools [18]. This partnership becomes crucial, especially when considering the ethical implications of AI applications in healthcare.

Using AI Tools to Craft Medical Illness Scripts and Exam Questions

ChatGPT is a deep learning-based language model trained on vast amounts of text data to generate human-like responses. While it does not store information between interactions, it can generate medical examination questions and illness scripts based on patterns learned during its training. Several studies have demonstrated its ability to produce such content. In the study by Yanagita et al., three physicians evaluated the AI-generated illness scripts for 184 diseases using a grading scale: "A" for sufficient, "B" for partially lacking but acceptable, and "C" for deficient. The results showed that GPT-4 successfully generated complete illness scripts for all diseases, with 56.0% rated "A," 28.3% rated "B," and 15.8% rated "C." This study concluded that GPT-4 could promptly create useful illness scripts [26]. In the study by Hudon et al., SCTs generated by ChatGPT were compared with those created by clinical experts and found that respondents found it challenging to distinguish between the AI-generated scripts and those generated by clinicians. Although ChatGPT demonstrated its capability to produce SCTs aligned with the authors ' predetermined clinical criteria, some outputs were criticized for oversimplifying or caricaturing scenarios [27]. Hudon et al. underscored ChatGPT's potential as a tool for writing SCT but highlighted the need for further refinement to ensure clinical accuracy and educational effectiveness [27].

Klang et al. generated 210 MCQs using GPT-4, which were then reviewed by physicians unaware of their origin. Of these, only one question was classified as "false," and 15% required some revisions. The study concluded that generative AI is a valuable supplementary tool for developing medical exam questions, provided specialist oversight exists [28]. Similarly, Cheung et al. used ChatGPT to generate 50 MCQs for medical examinations, finding their quality comparable to those written by university professors [29]. Therefore, generative AI tools like ChatGPT are highly efficient for producing high-quality medical scripts and clinical vignettes more quickly than their human counterparts. However, despite their advantages, these tools are prone to errors and should be used as adjunctive resources, with thorough review by experienced medical professionals.

Using AI to Enhance Learning, Assessment, and Diagnostic Capabilities

AI holds significant promise as a learning aid, particularly in training simulations. Wang et al. demonstrated this by using a virtual patient simulator that allowed students to interact using natural language. Students who engaged with this system showed improvements in clinical skills, as assessed automatically by the simulator based on their performance [39]. Similarly, Merritt et al. found that residents using AI-driven simulated case presentations, with AI acting as a primary care physician, reported enhanced communication skills and confidence in future interactions [45]. Hershberger et al. further supported this, showing that AI analysis of resident interview transcripts had moderate agreement with human raters (Kappa = 0.52) and a narrower range of agreement (Kappa = 0.313-0.658) when evaluating reflective statements, open-ended questions, closed-ended questions, and readiness-to-change assessments [48]. These findings underscore AI's potential to augment learning outcomes and assessment capabilities in educational settings, particularly in healthcare training. Overall, the results were promising, with AI tutors showing partial agreement with human decision-making and significant improvements across various learner groups in medicine: medical students, residents, and those in CME.

AI’s utility can extend beyond language skills into diagnostics. Cheng et al. applied AI to generate fracture probability maps on X-rays, significantly enhancing students' accuracy in identifying hip fractures, even without AI assistance [38]. Additionally, McFadden and Crim reported that primary care physicians at a CME conference who used AI-driven simulations showed marked improvement in diagnosing case vignettes, indicating enhanced diagnostic capabilities [52]. Suebnukarn and Haddawy developed the collaborative intelligent tutoring system, an automated tutor for small groups, which made choices consistent with human tutors 62%-83% of the time [43]. Another AI tutor, CIRCSIM-Tutor (Illinois Institute of Technology, Chicago, IL, and Rush College of Medicine, Chicago, IL), demonstrated significant learning gains in students, particularly in problem-solving questions, as evidenced by pretest and posttest comparisons [44]. While these studies report that AI improved diagnostic skills, communication skills, and radiograph interpretation for medical professionals, many studies were limited by the absence of control groups and relied on self-assessment for measuring outcomes.

Need for Structured AI Education in Medicine

Understanding the basic principles of AI is essential for its effective use, yet this knowledge may be limited, given the technology's relatively recent development. One study revealed that 66% (n = 29) of the surveyed faculty had utilized ChatGPT [55]. Understanding the normal operating parameters and limitations of AI is essential to ensure its effective application and to prevent undesired outcomes. Brief introductions to AI have proven beneficial in various contexts and cultures. For instance, a 2022 study of students in Lebanon found that those who received AI education from their medical school were more knowledgeable about AI than those who did not [90]. Another 2022 study in Germany demonstrated that an online class improved students' self-perceived AI readiness [86]. Similarly, a 10-hour pilot class for medical students at the University of British Columbia helped students better understand ML concepts [83,86]. Most learners reported increased confidence and satisfaction with AI-related courses [77-88]. These studies underscore the importance of AI education and indicate a positive trend toward embracing AI in the medical field to enhance both learning and clinical practices. Most students who completed the elective proposed by Abid et al. reported high satisfaction and increased confidence and understanding of AI [81]. The authors recommended incorporating such initiatives into medical school curricula to enhance AI literacy among future physicians [81]. However, caution is advised when relying on self-reported competence, as definitions can vary, and self-assessed competence may not always align with objective measures [91].

Limitations

The study on AI in medical education reveals several key limitations. Many studies included were qualitative, making it difficult to generalize the results or draw definitive conclusions. Additionally, confidence intervals or p values were often unavailable, and while the authors tried to quantify the findings, most studies remained qualitative by nature. This is likely due to the relatively recent introduction of AI models like ChatGPT to the public, meaning the existing literature consists of smaller, less structured studies. Many studies also lacked control groups or relied on self-assessment, which could introduce bias. The review was also limited to studies from PubMed, leaving out potentially relevant research from other sources. Furthermore, only studies in the English language were included, and unpublished data or preprints, which may provide more robust or cutting-edge conclusions, were not considered. ChatGPT's inability to interpret questions involving tables, graphs, or images restricted evaluations to text-based questions only. While AI showed promise in generating medical content, human oversight was often necessary to correct errors in the outputs. Additionally, AI faced challenges in ethical decision-making, emphasizing the need for human oversight.

## Conclusions

AI holds great promise as a learning aid and teaching tool in medical education. While research on its application in this field is encouraging, further studies with objective assessments and control groups are necessary to draw more definitive comparisons. Meanwhile, techniques like fine-tuning can enhance AI accuracy for specific tasks. Before integrating AI into teaching or personal study, educators and students should understand the principles, capabilities, and limitations of ML. Although comfort levels with AI vary among different groups, brief introductory sessions have been shown to effectively reduce unease when encountering this technology.
